# Nurse prescribing of medicines in 13 European countries

**DOI:** 10.1186/s12960-019-0429-6

**Published:** 2019-12-09

**Authors:** Claudia B. Maier

**Affiliations:** 1 Department of Healthcare Management, Technische Universität Berlin, H 80, Straße des 17. Juni 135, 10623 Berlin, Germany; 2 Center for Health Outcomes and Policy Research, University of Pennsylvania School of Nursing, Claire Fagin Hall, 418 Curie Blvd, Philadelphia, PA 19104 United States of America

**Keywords:** Health professionals, Nurses, Prescribing, Medications, Laws, Reforms, Advanced practice nursing (APN), Task shifting, Scope of practice

## Abstract

**Background:**

Nurse prescribing of medicines is increasing worldwide, but there is limited research in Europe. The objective of this study was to analyse which countries in Europe have adopted laws on nurse prescribing.

**Methods:**

Cross-country comparative analysis of reforms on nurse prescribing, based on an expert survey (TaskShift2Nurses Survey) and an OECD study. Country experts provided country-specific information, which was complemented with the peer-reviewed and grey literature. The analysis was based on policy and thematic analyses.

**Results:**

In Europe, as of 2019, a total of 13 countries have adopted laws on nurse prescribing, of which 12 apply nationwide (Cyprus, Denmark, Estonia, Finland, France, Ireland, Netherlands, Norway, Poland, Spain, Sweden, United Kingdom (UK)) and one regionally, to the Canton Vaud (Switzerland). Eight countries adopted laws since 2010. The extent of prescribing rights ranged from nearly all medicines within nurses’ specialisations (Ireland for nurse prescribers, Netherlands for nurse specialists, UK for independent nurse prescribers) to a limited set of medicines (Cyprus, Denmark, Estonia, Finland, France, Norway, Poland, Spain, Sweden). All countries have regulatory and minimum educational requirements in place to ensure patient safety; the majority require some form of physician oversight.

**Conclusions:**

The role of nurses has expanded in Europe over the last decade, as demonstrated by the adoption of new laws on prescribing rights.

## Background

The right to prescribe medications has for long been reserved to the medical profession only. This situation has changed, with an increasing number of countries worldwide having introduced reforms to authorise nurses to prescribe certain medications [1–6]. The United States of America (US) and Canada have a long tradition with nurses working in advanced practice roles, which includes the right to prescribe medicines [7–9]. Common drivers that led to nurse prescribing reforms include physician shortages (e.g. in rural areas), the rise in chronic conditions, more interprofessional team work and an increase in nursing education at higher educational institutions [1, 3, 10]. Introducing new roles for nurses has been referred to as a disruptive innovation in healthcare [11]. Advanced nursing roles have shown to have implications not only for the nurses and the teams in which they practice and are influenced by policies and regulatory mechanisms [12, 13]. For instance, changes to scope-of-practice laws and policies on advanced educational programmes have shown to facilitate the uptake of nurse prescribing and other advanced practice nursing roles [12–14].

Nurse prescribing refers to the official right granted to nurses to prescribe certain medications [4, 15]. The extent of nurse prescribing depends on several factors: first, the groups of nurses authorised to prescribe, which can range from small, highly specialised groups of nurses to all professional nurses; second, the type of medications that nurses are allowed to prescribe, which can range from all medicines to a restricted set; and third, the overall legal responsibility, ranging from independent prescribing to a delegated model under the supervision of a physician [15–17]. Nurse prescribing has been shown to be comparable to physicians’ prescribing practices, as measured by the number of medications prescribed as well as the types and doses of the medications chosen [6, 18, 19]. Patients were similarly satisfied or more satisfied with nurses than physicians [18]. A Cochrane systematic review showed that nurses were as effective in prescribing medications as physicians for a range of conditions including chronic diseases [19].

An international survey in ten high-income countries (Australia, Canada, Finland, Ireland, the Netherlands, New Zealand, Spain, Sweden, the United Kingdom and the US) showed that the educational and legal conditions varied across countries [1]. Among high-income countries worldwide, Australia, Canada, New Zealand and the US are commonly cited as having implemented laws granting the prescribing of a wide range of medicines to advanced practice nurses (APN) with a Master’s degree [1, 2]. APN in these countries are authorised to prescribe all or almost all medicines within their specialty. However, differences exist in the regulation of nurse prescribing, namely if collaborative agreements with physicians are required by law [6, 20, 21]. In addition, in Australia, Canada and New Zealand, there have also been recent developments to grant registered nurses limited prescribing rights [21, 22].

In Europe, the United Kingdom (UK) has a long experience with nurse prescribing [6], where two models of nurse prescribing originated [15, 23], independent and supplementary nurse prescribing. While the former model grants the nurses the authority to issue prescriptions independently including initial prescribing of a medicine for the first time, the latter model refers to the continued prescribing after a diagnosis and treatment plan has been made by a physician [15, 23]. Research on nurse prescribing reforms has been limited and mostly focused on selected topics and individual countries, such as in the UK and Ireland on prescribing practices and safety considerations [24, 25] as well as factors enabling implementation of reforms in Ireland [26]. Recently, research in the Netherlands has evaluated task shifting between physicians and nurse specialists with generally positive results [27, 28]. Research in the Netherlands also assessed physicians’ and nurses’ views of introducing nurse prescribing [23] as well as prescribing practices of nurses in hospitals, which were shown to be influenced by national policies as well as individual hospital governance structures [29]. From an international, cross-country perspective, research on nurse prescribing reforms and the extent of prescribing authority of medicines has been scarce. One exception is a review by Kroezen et al. [1]. The review covered 10 countries of which six belong to the European Union (Finland, Ireland, the Netherlands, Spain, Sweden, and the United Kingdom). It showed that at the time of data collection, three countries (Finland, Spain, the Netherlands) were in the process of introducing legislation, suggesting an update of the current state of these reforms is warranted. Moreover, no study to date has systematically covered all countries belonging to the EU’s single market.

The purpose of this study was to analyse which countries in Europe have introduced nurse prescribing reforms, the extent of prescribing and regulations in place. Covering all countries belonging to the EU’s single market is of relevance for its free movement principle across borders which also applies to health professionals. For the nursing profession, the first-level education as professional nurse is automatically recognised, yet no nurse specialisations are covered under the EU regulation [30, 31].

## Methods

The study was based on an expert survey to identify which countries in Europe have implemented reforms on nurse prescribing of medicines and, in those countries with reforms, an analysis of the extent of prescribing authority, regulatory and oversight requirements.

### Expert survey to identify countries with prescribing reforms

An expert survey was conducted in 39 countries (TaskShift2Nurses Study) in 2015 covering all EU Member States and countries belonging to the EU’s single market, plus Australia, Canada, New Zealand and the US. A total of 93 country experts participated (response rate 85.3%). The sampling was based on two interrelated strategies: snowballing and network strategies to counteract the weaknesses associated with snowballing alone, which has been associated with selection bias or too homogeneous groups [2]. The selection process of the country experts followed two stages: first, five international health workforce experts were identified and contacted to provide names of country experts. In a second step, the suggested names were reviewed and contacted if in accordance with a list of pre-defined criteria (e.g. senior position related to nursing either in academia, at national nursing association, Ministry of Health, relevant publications on the topic, proficiency in English). More details about the methodology are available elsewhere [2]. Among the questions covered in the survey were if nurses were authorised to prescribe medications and related reforms implemented or ongoing, as of 2015.

### Analysis of country reforms on nurse prescribing

Of those countries identified with reforms on nurse prescribing, an in-depth analysis of laws and policy documents was performed as part of an Organisation for Economic Cooperation and Development (OECD) study. Country experts were contacted in 2016 for the purpose of the OECD study on nurses in advanced roles of which prescribing of medicines was one example [4]. A total of 25 country experts from those countries with nurse prescribing reforms implemented or ongoing were contacted and 21 participated (response rate 84%). They provided additional information on the laws, the extent of prescribing rights (e.g. for which medicines and health conditions), educational, regulatory and oversight requirements in the countries. For the purpose of this paper, the country experts were again contacted in 2019 to inquire if the information provided in 2016 was still up to date. A total of 15 experts participated in the 2019 round (response rate 60%) and provided updates to pre-filled forms based on the 2016 data collection. In five cases, there was a lack of clarity on specific issues, which was then followed up via e-mail or phone calls and resolved through discussion.

In addition, a literature search was conducted with a search of the peer-reviewed (PubMed, CINAHL, Google Scholar) and grey literature (websites of International Council of Nurses, European Federation of Nurses, WHO, OECD, national Ministries of Health). Search terms covered a set of terms combined with Boolean operators on nurses (e.g. registered nurse, professional nurse, nurse specialist, Advanced Practice Nurse, Nurse Practitioner and other titles and combinations), prescribing (prescri*) and the names of the 28 EU countries plus Norway and Switzerland.

The analysis was based on thematic analyses of the laws, policy documents and other grey material, focussing on the content of the laws, by-laws and the educational and regulatory requirements if stipulated in other legal documents. The analyses were structured according to the groups of nurses authorised to prescribe, by medicines used for the prevention or treatment of major conditions to assess the extent of prescribing, differentiating between initial prescribing (IP) and continued prescribing (CP) only. Finally, additional requirements were analysed by country and groups of nurses, including mandatory registration requirements (e.g. in a registry), levels of physician oversight and other oversight requirements. The analysis was performed by the author using standardised word tables based on the survey responses, complemented with information from the laws and legal documents and fed back to the country experts as part of the 2019 round where experts were requested to provide updates of the information or confirm that the information was up to date.

## Results

As of 2019, 13 countries in Europe have laws on nurse prescribing in place, which apply nationwide in 12 countries (Cyprus, Denmark, Estonia, Finland, France, Ireland, Netherlands, Norway, Poland, Spain, Sweden, United Kingdom) and in one region in Switzerland in the Canton Vaud (Table 1).

**Table 1:** Nurse prescribing laws, years of adoption and groups of nurses authorised to prescribe medications, 13 countries

Year first adopted	Country	Regulation	Group(s) of nurses authorised to prescribe (qualification requirements)
1992	UK	Medicinal Products: Prescription by Nurses etc. Act 1992	(i) Independent prescribers (full prescribing rights since 2006; including certain controlled drugs since 2012, course on independent nurse prescribing required (NMC approved post-registration prescribing programme e.g. v200 or v300 course, length varies, e.g. 30–45 UK credits, typically 6 months; as of September 2019, new standards apply), this includes Community Practitioner Nurse Prescribers (CPNP) with prescribing rights according to a formulary (e.g. V100 or V150, e.g. 10 UK credits), (ii) Supplementary prescribers since 1992 (limited prescribing rights, approved course as a supplementary prescriber)
1994	Sweden	Law	RN (Bachelor from university or university college, 180 ECTS)
2002	Norway	Law	Public health nurse specialisation (Bachelor, plus 60 ECTS)
2007	Ireland	Pharmacy Act, Statutory Instrument No. 201/2007 on Medicinal Products	Nurse prescriber: RN plus additional, approved educational programme (range 20 to 40 ECTS for nurse prescriber certificate or higher if Master APN programme)
2009	Denmark	Order 1219 of 11/12/2009 delegation of reserved procedures	RN (Bachelor, of which 30 ECTS “cluster” on medical treatment, including prescribing)
2010	Finland	Decree 1088/2010 on Prescriptions	Nurse prescriber: RN (Bachelor, with postgraduate education in prescribing (45 ECTS)
2012	Netherlands	Decree, December 21/2011	Nurse specialist (*Verpleegkundig Specialist*) (Master APN, 120 ECTS)
2012	Cyprus	Nursing and Midwifery Law, Annex III, 2b) V	APN nurse (Master APN degree with specialisation in Midwifery, ICU, Mental Health, Oncology, Community health). Advanced pharmacology (6 ECTS) is part of the Master APN programme
2014	Netherlands	Decree of 2014	Diabetes, lung, oncology nurses (Bachelor, and completion of pharmacotherapy course at a University of Applied Science, e.g. for diabetes and lung nurses, curricula comprise minimum of 2.5 ECTS on prescribing
2015	Poland	Ordinance of 28/10/2015 on prescriptions issued by nurses and midwives	Two-tiered: RN with (i) Master and (ii) Bachelor’s degree
2015	Spain	Royal Decree 954/2015 of 23 October	RN (Bachelor with min. 1 year work experience; or additional training in prescribing if < 1 year experience)
2016	Estonia	Health Services Organization Act 2016	Family nurse (*pereõde*) if working with a family doctor (*pereast*) with completed training of 120 hours (clinical pharmacology, approved by the Agency of Medicines)
2017	France	Act 2016-41 on the modernisation of the health system	Nurse (“Medical auxillaires”)
2017	Switzerland (Vaud)	Article 124b of Public Health Act 800.01	Nurse specialist (Master) (“*Infirmier practicien spécialisé”*)

*ANP* advanced nursing practice, *APN* advanced practice nurse, *CPD* continuous professional development, *RN* registered nurse, sources: [16, 32–45]

The four nations of the UK and Sweden were the first countries in Europe which introduced prescribing rights dating back to 1992 and 1994, respectively. Countries that followed were Norway, Ireland and Denmark in 2002, 2007 and 2009. Since 2010, eight additional countries (including the Canton Vaud, Switzerland) have authorised specific groups of nurses to prescribe certain medications. All have adopted laws to officially grant nurses prescribing rights, in some cases followed by the adoption of decrees to define under which conditions nurses are allowed prescribe, for what medications and the regulatory requirements.

The countries vary considerably in the groups of nurses that are authorised to prescribe and their educational requirements: a first group consists of countries in which the prescribing skills and competencies are part of the nurses’ education, e.g. Master’s or Bachelor’s degrees or specific nurse specialisations. Hence, the prescribing skills and competencies are directly integrated in these curricula, e.g. ANP Master nurses in Cyprus, Nurse Specialists with APN degree in the Netherlands, Public Health Nurses in Norway, nurses with Master’s and Bachelor’s degree in Poland, family nurses in Estonia; and nurses with a Bachelor’s degree in Spain. Since courses on pharmacology and pharmacotherapy are routinely part of these curricula, it was only in a few cases possible to single out how many European Credit Transfer Systems (ECTS) points are allocated specifically on skills and competencies related to the prescribing of medicines. For instance, in Cyprus, the curriculum of APN Master-level nurses comprises advanced pharmacology and pharmacotherapy of approx. six ECTS. In Denmark, prescribing is taught as part of a 30 ECTS “cluster” on medical treatment and therapy. In the Netherlands, RNs with a specialisation in diabetes and RNs with a specialisation of lung care are required to have a minimum of 2.5 ECTS on prescribing in their curriculum [32].

Second, some countries have set up a separate educational pathway specifically on nurse prescribing, hence, *additional* educational courses which lead to a title as nurse prescriber or similar. The countries are Finland, Ireland and the UK. The UK has introduced two models of prescribing, independent and supplementary prescribers. Ireland and Finland have established one role. Educational requirements vary, they range between typically 30 to 45 UK credits in the UK, 20 to 40 ECTS in Ireland and mandatory 45 ECTS in Finland. In 2019, the UK Nursing and Midwifery Council has published new standards for the post-registration programmes which apply since September 2019 [46].

France and the Swiss Canton Vaud are in the process of implementing the 2017 laws on nurse prescribing, pending the agreement of formularies and other minimum requirements as a prerequisite for nurses to officially being authorised to prescribe medicines.

### Extent of prescribing rights

Table 2 shows the extent of prescribing rights by country. The countries vary considerably in the number of medicines which nurses are officially allowed to prescribe, for which diseases and the type of prescribing. The type of prescribing refers to initial and continued prescribing, of which the former is the right to newly prescribe medicines, whereas the latter refers to follow-up prescriptions after the diagnosis and first prescription has been issued by a physician.

**Table 2:** Extent of nurse prescribing, by group(s) of nurses and country

		Prescribing rights by major areas and conditions (IP = initial prescribing, CP = continued prescribing only)					
Country	Name/title of nurses	Vaccines	Contraceptives	Chronic conditions	Acute illnesses	Pain medication	Other
Full prescribing rights (within specialty)							
Ireland	Nurse prescriber^1^	IP	IP	IP	IP	IP	IP
Netherlands	Nurse Specialist^2^	IP	IP	IP	IP	IP	IP
UK	Independent prescriber^1^	IP	IP	IP	IP	IP	IP
Limited prescribing rights							
Denmark	Registered Nurse^3^	CP	CP	CP	CP	CP	CP
Estonia	Family nurse (*pereõde*)	–	CP (hormonal contraceptive)	CP (diabetes, hypertension)	CP (acute cystitis, nitrofurantoiin)	–	–
Finland	Nurse prescriber	IP (influenza, hep., varicella^)	IP (hormonal contraceptive^#)	CP (asthma, dyslipidemia, T2D, hypertension)^	IP (pharyngitis)^, CP (UTI)^	IP (e.g. local anaesthetics)^	–
Netherlands	Diabetes, lung, oncology nurses	–	–	IP (diabetes, oncology, lung diseases)	–	IP (oncology)	–
Norway	Public Health Nurse	IP	IP (including IUD)^a^	–	–	IP (adrenaline for allergic reaction, local anaesthetics)	IP (sterile equipment for IU, implants, STD kits)
Poland	RN (Master)	–	IP (gynaecological drugs)	IP (asthma, e.g. bronchodilators)	IP (throat, ear, sinus, UTI)	IP (analgesics, locally acting anaesthetics)	IP (anti-emetics, anti-parasitic, IV infusion fluids)
Poland	RN (Bachelor) ^4^	–	CP (as above)	CP (as above)	CP (as above)	CP (as above)	CP (as above)
Spain	RN (Bachelor)^5^	IP (according to vaccination schedule)	IP (emergency contraception)	CP	CP	CP	IP (OTC)
Sweden	RN (Bachelor)	–	–	–	IP (throat, mouth, dermatological disease, GI, UTI)	IP (pain management)	–
UK	Supplementary prescriber	CP	CP	CP	CP	CP	CP

Cyprus, France, Switzerland (Vaud): not listed because no information available on the medicines/formulary, *IP* initial prescribing (prescribing right of a new medicine/product), *CP* continuous prescribing (follow-up prescribing after first prescription issued by physician), *n/r* not reported (in the law/regulations, “-“ not authorised to prescribe any medicine/product in the area, *n/a* no information available, *OTC* over-the-counter medicines, *Hep*. hepatitis, *UTI* urinary tract infection, *GI* grastrointestinal. ^1^Initial prescribing rights of all medicines falling within nurses’ specialty, restrictions and additional requirements apply to controlled drugs (e.g. UK: controlled drugs except for cocaine, dipipanone or diamorphine for treating addiction), ^2^Netherlands = initial prescribing rights of all medicines falling within nurses within nurse specialists’ specialty, Cyprus = details on the types of medicines/substances not (yet) specified in law; ^not for children under the age of 12, #not for women under age 35, ^a^only for women over 16 years of age, ^3^Denmark = continued prescribing according to local frame prescriptions and in a delegated model, ^4^Poland: prescribing rights according to formulary of 12 groups of medicines, ^5^Spain: prescribing rights granted to all RN with minimum 1 year work experience; for RN with less than 1 year work experience, additional training required

Three of the 13 countries have granted full or nearly full prescribing rights to a specific group of nurses. The countries are Ireland (nurse prescribers), the Netherlands (nurse specialists) and the UK (independent nurse prescribers). These groups of nurses are by law allowed to prescribe any medicine within their specialty. In the UK and Ireland, the extent of prescribing was expanded gradually over time. In the UK, the law was changed in 2006 authorising independent prescribers full access to the British National Formulary granting the same prescribing rights as for physicians, and subsequently in 2012 to cover certain controlled drugs [16]. In Ireland, nurse prescribers can prescribe a full set of medicines since 2007, including certain controlled drugs. In the Netherlands, an initially time-limited law was introduced in 2012, linked to a nationwide evaluation. The law granted nurse specialists with a Master’s degree APN full prescribing rights within their specialty. After a generally positive evaluation, the time-limited nature of the law was changed to unlimited duration in September 2018 [27].

In the remainder countries, the extent of prescribing is limited, either in the number of medicines that nurses are by law allowed to prescribe or in the type of prescribing, allowing primarily or exclusively continued prescribing. In the Netherlands (Bachelor nurses with a specialisation in either diabetes, lung or oncology), Norway (public health nurses), Poland (Master level) and Sweden (Bachelor level), nurses are authorised to initially prescribe certain medicines from a limited set of medicines (Table 2). In the Netherlands, the three Bachelor-level nurse specialisations are authorised to initially prescribe a limited number of medicines within their specialty (prescription-only medications), after a diagnosis has been made by a physician, and as specified within protocols and standards. In Norway, public health nurses work in child health clinics and frequently in schools or youth health centres where they provide health counselling including on sexual health and prescribe contraceptives [47]. Public health nurses can officially prescribe all contraceptives for all women aged 16 years and over. In one study, public health nurses wrote more prescriptions than physicians for young women aged 17–18 years [47].

A mix of initial and continued prescribing exists in Finland and Spain; it includes initial prescriptions of vaccines and contraceptives in Finland and Spain and follow-up medications for highly prevalent chronic and acute conditions. In Denmark (Bachelor), Estonia (family nurse), Poland (Bachelor) and the UK (supplementary prescribers), nurses are authorised to perform continued prescribing, according to patient management plans and in a delegated model.

No information on the details of prescribing rights was identified for Cyprus, France and the Canton Vaud (Switzerland). In Cyprus, there is no information provided in the 2012 law on what medicines the Master APN nurses are allowed to prescribe. The law states that medicines from a list can be prescribed by nurses, but with no further information. In France and Vaud, due to the adoption of the laws in 2017, developments are ongoing to specify which medications nurses will be able to prescribe.

### Regulatory and oversight requirements

All countries have defined regulatory requirements as a pre-condition for nurses to prescribe medications (Table 3). The reason is the highly specialised nature of prescribing. The majority of countries require some form of additional registration as a prescriber in a registry or a prior authorisation by a competent authority. Several countries added additional regulatory requirements, e.g. in Ireland, UK and Finland, nurse prescribers receive a unique ID number to facilitate the identification of who prescribes what medication. In addition, most countries require some form of official authorisation, contract, collaboration, agreement or official supervision by an individual physician (Denmark, Estonia, Finland, Ireland, Spain, UK).

**Table 3:** Regulatory and oversight requirements

Country	Group of nurses	Registration/authorisation	Physician oversight officially required? (e.g. collaborative agreement)	Other regulatory requirements (e.g. protocols, employer-level requirements)
Cyprus	APN	Yes (authorisation from competent authority)	n/r	n/r
Denmark	RN	No	Formal collaboration with a physician	Individual frame prescriptions with physician required
Estonia	Family nurse (*pereõde*)	Yes (Health Board, Healthcare Workers’ Registry)	Required to work with a family physician	No
Finland	RN	Yes	Authorisation by a physician	Employment with municipal health center; ID number with National Supervisory Authority for Welfare and Health
Ireland	Nurse prescriber	Yes (An Bord Altranais)	Collaborative agreement with a physician required	Employment in healthcare setting, personal identification number (PIN)
Netherlands	Nurse specialist	Yes (nurse specialist registry)	No	No
	Diabetes, lung, oncology specialist nurses	No (not legally required, but voluntary registration possible)	No	Following protocols and after initial diagnosis by a physician
Norway	Public health nurse	Yes (National registry of healthcare professions)	No	Formal documentation of prescriptions (as any other profession), listed in central registry for prescriptions logs
Poland	RN (Master)	No	No	No
	RN (Bachelor)	No	Continuous prescribing only, after initial diagnosis by physician	No
Spain	RN (Bachelor)	Yes (regions in charge of issuing certificates on prescribing)	Yes, physician supervision	For certain medicines, prescribing rights to be defined in protocols and clinical guidelines, to be developed by the Medical and Nursing Councils and MoH
Sweden	RN (Bachelor)	Yes (prescribing code at Board of Health and Welfare)	No (e.g. if employed as district nurse, works independently)	Employment with county council, primary, home health or elderly care
UK	Independent prescriber	Yes (annotation in registry as independent prescriber)	Prescribing partnership with physician required	Support from employing organisation, personal identification number
	Supplementary prescriber	Yes (annotation as supplementary prescriber)	Clinical management plan, prescribing partnership with physician	Support from employing organisation, ID number, continued prescribing of medicines listed in individual clinical management plan agreed between patient, physician and supplementary prescriber

*APN* advanced practice nurse, *MoH* Ministry of Health, *n/r* not reported (in law/regulation), *RN* registered nurse. France, Canton Vaud (Switzerland): no information available

## Discussion

This study shows that in Europe, certain groups of nurses are officially authorised to prescribe medications in 13 countries (nationwide in 12 countries and in one region in Switzerland, Canton Vaud). The majority of the reforms have been introduced over the past decade. Since 2010 alone, eight of the 13 countries newly introduced nurse prescribing (Finland, Netherlands, Cyprus, Estonia, Poland, Spain, France and the region Vaud in Switzerland). Hence, nurse prescribing has been a recent development in several countries in Europe. The extent of prescribing rights varies considerably, with three countries (Ireland, Netherlands, UK) granting certain groups of nurses (nurse prescribers, nurse specialists, independent nurse prescribers, respectively) almost full prescribing authority within their specialty. In the other countries, the number of medications is restricted, defined in a formulary or can be prescribed only after an initial prescription has been made by a physician. All countries have regulated the conditions under which nurses are allowed to prescribe; the majority require additional registration in the prescribing function, some form of physician oversight and other measures to ensure patient safety.

The study faces several limitations. First, it has exclusively focused on nurse prescribing; however, some of the countries have also introduced prescribing for other non-medical professions, such as midwives or pharmacists. Second, while the article provides an overview of the groups of nurses and extent of prescribing rights, the exact types and doses of medicines were not covered in-depth and should be investigated in future research. Third, information on educational requirements for nurse prescribing was difficult to obtain, particularly for countries where nurse prescribing is integrated in basic or advanced educational programmes.

The findings are largely consistent with previous research [1, 3, 4] and provide an update with more European countries covered and recent reforms included. While several studies in the past have focused on Anglo-Saxon countries, with Ireland and the UK frequently covered in research [10, 15], this study has enabled to cover more European countries. This study shows that the extent of nurse prescribing varies considerably across the countries studied and for specific groups of nurses within three countries (Netherlands, Poland, UK), which is consistent with the previous literature (ibid). Most countries in this study restricted prescribing rights to a list of medicines with regulatory requirements including physician oversight. In previous research, due to the differences in country coverage, which covered six European countries (Finland, Ireland, Netherlands, Spain, Sweden, UK) and Australia, Canada, New Zealand and the United States, the findings showed higher levels prescribing rights and independence in prescribing, e.g. for advanced practice nurses [1, 17].

The findings of this study show that there are high variations in the educational requirements, ranging from Bachelor level (e.g. Denmark, Spain, Sweden) to Master level degrees (e.g. Cyprus, Netherlands, Poland). There was no obvious link between the extent of prescribing rights and the length and level of training across the countries. In the three countries with almost full prescribing rights, educational requirements also varied. In the Netherlands, nurse specialists are required to hold a Master’s degree (120 ECTS) as a prerequisite to prescribe medicines. In Ireland and the UK, the competencies are taught in prescriber courses that are approved by the competent authorities, but the length varies and a Master’s degree is not required. There is a paucity of research linking the educational requirements with prescribing practices, the quality of prescribing and patient outcomes. While previous research has analysed the association of higher nurse education (e.g. higher proportion of Bachelor-level nurses or a higher proportion of professional nurses among all nurses) with improved patient outcomes and mortality [48, 49], no research was identified on the association between qualification and prescribing outcomes.

The reforms introducing nurse prescribing in 13 countries span different health systems, educational systems and geographic locations across Europe, including predominantly social health insurance (France, Netherlands) and Beveridge models (e.g. UK, Ireland). The reasons of introducing these reforms and new laws have not been systematically investigated across countries. Previous research suggests several potential drivers, including increasing patient needs and volume, higher education of nurses, higher workloads among physicians, inefficient division of work and high costs, among others [17]. Other research identified the roles, skills and competencies of individual prescribers, professional boundaries, organisational and institutional contexts as potential drivers or barriers [12, 13, 50]. In Ireland, several facilitating factors were identified as having contributed to the successful implementation of nurse prescribing: strong advocacy by the nursing profession, planning for nurse education and practice, support for multiprofessional teams and supportive government action [26]. Overall, it appears that the interplay of different factors, including nurses’ prescribing skills and education, organisational factors as well as government, stakeholder and policy support determine the implementation process. Moreover, reforms appear to take time. In the three countries in our study that grant almost full prescribing rights to specific groups of nurses (Ireland, Netherlands, UK), the policy process was lengthy and evolved over time. In Ireland and the UK, the extent of prescribing rights were initially limited and gradually expanded over time in line with generally positive evaluations [15].

One driver often referred to in Europe is the higher education of nurses in line with the Bologna cycle [17, 51, 52]. This may explain the occurrence of informal prescribing practices among nurses which paved the way towards formalising nurse prescribing, as reported in the Netherlands and Spain [53, 54]. In the Netherlands, this argument was strong in the policy debate on whether to introduce a law; controversially debated between the medical and nursing associations [53]. Overall, there is a lack of systematic cross-country research on the role of various influencing factors acting as barriers or facilitators to the introduction of nurse prescribing. In particular, more research is needed on why nurse prescribing laws have been adopted in the 13 European countries and not others.

In three of the 13 countries, the implementation of the laws is ongoing, pending the adoption of regulatory decrees and capacity building in education. France and the Canton Vaud have in common that the laws have been recently adopted in 2017. In Cyprus, the year of adoption was 2012. In Cyprus, there is no evidence of nurse prescribing officially taking place in practice, as no individual request has been made to authorise prescribing rights.

The fact that to date 13 countries belonging to the EU’s single market grant certain groups of nurses prescribing rights demonstrates that further cross-country research is required from an EU perspective. Research should focus on commonalities and differences in prescribers’ education across Europe, country variations in prescribing practices as well as the extent of prescribing rights and outcomes on specific patient groups. Moreover, with the increase in health professional mobility across Europe, including nurses, a timely monitoring of nurses with prescribing rights is warranted as to avoid skills mismatches when moving borders [55–57].

## Conclusions

A total of 13 countries in Europe have laws on nurse prescribing in place, of which the majority adopted laws over the past decade, suggesting a recent trend expanding the roles of nurses in these countries. The extent of prescribing rights varies considerably, with three countries granting full prescribing rights, whereas the majority of countries have restricted prescribing rights, particularly those with recent reforms. From an EU perspective, future cross-country research is required to monitor the education, prescribing practices and mobility patterns of nurses with prescribing qualifications.

## Data Availability

The laws and other documents are publicly available.

## Abbreviations

APN: Advanced practice nurse
CP: Continued prescribing
CPD: Continuous Professional Development
CPNP: Community Practitioner Nurse Prescribers
Dir.: Directive
ECTS: European Credit Transfer System
EU: European Union
GI: Gastrointestinal
Hep.: Hepatitis
ICU: Intensive care unit
ID number: Identification Number
IP: Initial prescribing
IUD: Intrauterine device
MoH: Ministry of Health
n/a: No information available
n/r: Not reported
NMC: Nursing and Midwifery Council
OECD: Organisation for Economic Cooperation and Development
OTC: Over-the-counter medicines
PHC: Primary health care
PHN: Public health nurse
RCN: Royal College of Nursing
RN: Registered nurse
STD: Sexually transmitted disease
T2D: Type 2 diabetes
UK: United Kingdom
US: The United States of America
UTI: Urinary tract infection
